# Natural Variation and Domestication Selection of *ZmPGP1* Affects Plant Architecture and Yield-Related Traits in Maize

**DOI:** 10.3390/genes10090664

**Published:** 2019-08-30

**Authors:** Pengcheng Li, Jie Wei, Houmiao Wang, Yuan Fang, Shuangyi Yin, Yang Xu, Jun Liu, Zefeng Yang, Chenwu Xu

**Affiliations:** Jiangsu Key Laboratory of Crop Genetics and Physiology/Key Laboratory of Plant Functional Genomics of the Ministry of Education/Jiangsu Key Laboratory of Crop Genomics and Molecular Breeding/Jiangsu Co-Innovation Center for Modern Production Technology of Grain Crops, Agricultural College of Yangzhou University, Yangzhou 225009, China

**Keywords:** natural variation, maize, nucleotide diversity, domestication selection, *ZmPGP1* gene

## Abstract

*ZmPGP1*, involved in the polar auxin transport, has been shown to be associated with plant height, leaf angle, yield traits, and root development in maize. To explore natural variation and domestication selection of *ZmPGP1*, we re-sequenced the *ZmPGP1* gene in 349 inbred lines, 68 landraces, and 32 teosintes. Sequence polymorphisms, nucleotide diversity, and neutral tests revealed that *ZmPGP1* might be selected during domestication and improvement processes. Marker–trait association analysis in inbred lines identified 11 variants significantly associated with 4 plant architecture and 5 ear traits. SNP1473 was the most significant variant for kernel length and ear grain weight. The frequency of an increased allele T was 40.6% in teosintes, and it was enriched to 60.3% and 89.1% during maize domestication and improvement. This result revealed that *ZmPGP1* may be selected in the domestication and improvement process, and significant variants could be used to develop functional markers to improve plant architecture and ear traits in maize.

## 1. Introduction

Maize (*Zea mays* L.) is one of the most widely grown and important cereal crops, which plays a critical role in ensuring food security. Maize was domesticated from the wild grass teosinte more than 8700 years ago [1]. The domestication of maize went through two stages: domestication selection and subsequent genetic improvement (post-domestication selection) [2]. Strong directional selection had profound effects on the morphological structure of maize, and genetic improvement affected its productivity [3]. For example, from 2000 to 2014, the total maize production in the United States and China increased by 31% and 49% respectively, of which half could be attributed to genetic advances [4,5]. Human selection has profound effects on the genetic diversity for the genomic region under selection and target genes [3]. Genetic consequences during the domestication and breeding history will enable us to understand its important role on yield increase in the modern maize breeding.

Grain yield (GY) is a complicated quantitative trait and is mainly determined by three yield components: effective ear number, kernel number, and kernel weight [6]. Maize kernel and ear morphological traits are the most important factors determining grain yield. Kernel weight is mainly affected by kernel size, which is usually measured by kernel length (KL), kernel width (KW), and kernel thickness (KT). Ear length (EL), ear diameter (ED), and kernel row number (KRN) are important traits determining the kernel number [7]. Planting density is a major factor in determining the effective ear number. The increased maize productivity is predominantly due to higher planting density, resulting from the domestication and improvement of plant shoot architecture [4]. Plant architecture is influenced by aboveground phenotypes, such as plant height (PH), ear height (EH), and leaf number (LN). From 1930 to 2001 in the United States, maize ear height was reduced by 3 cm per decade, leaf angle became more upright, tassel branch numbers became averaged 2.5 fewer branches per decade, and leaf number increased from 12.2 in the 1930s to 13.8 in the 1970s [8]. Identification of genes associated with grain yield and plant architecture traits will be helpful for maize yield improvement.

Most plant and ear traits are quantitative traits, which are controlled by a large number of small effect quantitative trait locus (QTLs). Many QTLs related to yield components and plant architecture traits have been identified in several maize linkage populations. A total of 163 QTLs were detected for four ear traits in 10 different RIL populations, accounting for 55.4–82% of phenotypic variation [9]. In the same panel, approximately 800 QTLs with major and minor effects were identified for 10 plant architecture-related traits [10]. Martinez et al. [11] assembled a yield QTLome database, and 808 QTLs for GY and seven additional GY components of common interest in maize breeding from 32 mapping populations were used for meta-QTL analysis. A total of 84 meta-QTLs were projected on the 10 maize chromosomes [11]. A number of genes that affect plant and ear traits have been identified, such as *fea2*, *fea3*, the ramose genes and *KRN4* for kernel row number, *df3*, *df8*, *df9*, and *br2* for plant height, and *td1*, *bif2*, *ba1*, and *tsh4* for tassel morphology [12,13,14,15,16,17,18,19,20,21]. Numerous kernel and morphological traits have changed during maize domestication and improvement, and some key genes have been cloned. *Tb1* has been shown to be associated with maize branching [22], the teosinte allele *gt1* confers multiple ears per branch [23], and *tga1* was associated with kernel structure [24]. In addition, genome-wide selection signals during maize domestication and improvement were assessed, 484 domestication and 695 improvement selective sweeps were detected, and a number of genes with stronger signals for selection underlie major morphological changes [3].

Indole-3-acetic acid (IAA), an active form of auxin, is a key regulator of plant growth and development. *ZmPGP1* (*ABCB1* or *br2*) was firstly cloned using a Mu element, the mutant was characterized by compact lower stalk internodes and the plants showed semi-dwarf stalks [16]. *ZmPGP1* was an adenosine triphosphate (ATP) binding cassette (ABC) transporter which belonged to the multidrug resistant (MDR) class of P-glycoproteins (PGPs), and functioned as an efflux carrier in polar auxin transport. The protein had two transmembrane domains that provide the translocation pathway of auxin and two cytoplasmic nucleotide-binding domains that hydrolyse ATP and drive the transport reaction [25,26]. Different alleles of *ZmPGP1* have been shown to be associated with plant height, ear height, leaf angle, ear length, yield traits, and root development under aluminum stress [16,27,28,29,30,31]. Although several mutations of *ZmPGP1* have also been identified, the sequence polymorphism and natural variations of the gene have not been investigated. It is also unclear whether *ZmPGP1* exists as a signal of selection during maize domestication and improvement. In the present study, we re-sequenced *ZmPGP1* in 349 inbred lines, 68 landraces, and 32 teosintes, and aimed to: (1) examine the *ZmPGP1* nucleotide diversity between maize inbred lines, landraces, and teosintes, (2) identify natural variations in candidate genes associated with grain yield and plant architecture traits, and (3) examine the significant associations for their involvement in maize domestication and improvement.

## 2. Materials and Methods

### 2.1. Plant Materials and the Phenotypic Evaluation

A total of 349 inbred lines, 68 landraces, and 32 wild relatives were selected in this study [32]. The inbred lines were grown in the field in a randomized block design with two replicates in 2016, 2017, and 2018 in Sanya, Hainan Province (18°23′ N, 109°44′ E). Each inbred line was grown in a single row with 13 plants, 3 m in length, and 0.5 m between adjacent rows. Fifteen days after pollination, 6 plants in the middle of each row were selected to measure leaf number above the topmost ear (LNAE), plant height (PH), tassel branch number (TBN), and tassel main axis length (TMAL), the first leaf above the ear position leaf was selected to measure leaf angle (LA, the angle between the horizontal and the midrib of the leaf) and leaf width (LW). The measure method of plant architecture traits referred to are as described in Pan et al. (2017) [10]. After harvesting and drying, 3 well-developed ears were selected to measure ear traits, including ear grain weight (EGW), 100-kernel weight (HKW), ear diameter (ED), ear weight (EW), ear length (EL), kernel length (KL), kernel width (KW), kernel thickness (KT), and kernel number per row (KNR). The root and shoot traits at the seedling stage were determined by Li et al. [32] in a hydroponic system.

### 2.2. DNA Isolation and ZmPGP1 Resequencing

A modified cetyl trimethylammonium bromide (CTAB) method was used to exact genomic DNA from young leaves of each line at the seedling stage. The sequences of the *ZmPGP1* gene were sequenced by BGI (Beijing Genomics Institute) Life Tech Co. China using targeted sequence capture technology on the NimbleGen platform [33]. The genomic sequence of *ZmPGP1* (GRMZM2G315375) of the B73 inbred line was used as a reference for target sequence capture.

### 2.3. Analysis of Sequence Data

Multiple sequence alignment of the maize *ZmPGP1* gene was performed using MAFFT software and was further edited manually [34]. Using DNASP5.0 software [35], we analyzed single nucleotide polymorphisms (SNP) and allelic diversities across all tested lines. Two parameters, π and θ, were used to estimate the degree of polymorphism within the tested population. Tajima’s D [36], Fu and Li’s D*, as well as Fu and Li’s F* [37] statistical tests were used to test for neutral evolution within each group and each defined region. The sequence data and markers were shown in Dataset 1–2.

### 2.4. Marker–Trait Association Analysis in Inbred Lines

Genotyping-by-sequencing (GBS) was used to identify the genotypes of 349 inbred lines [32]. A total of 163,931 SNPs were obtained by filtering out markers with more than 20% of missing data and below 1% minor allele frequency. Three models were used to conduct marker–trait associations: (1) the K model, controlling for kinship, (2) the PCA + K model, controlling for both population structure (principal component, PC) and kinship, and (3) the Q + K model, controlling for both population structure (Q) and kinship. Principal component analysis (PCA) and kinship were calculated using Tessel5.0, and Q was calculated by admixture. A total of 499 *ZmPGP1*-based markers with minor allele frequency (MAF) ≥0.05 were selected for association analysis in 349 inbred lines, and the *p* value threshold was set at 2.00 × 10^−3^ (0.5/499).

## 3. Results

### 3.1. Sequence Polymorphisms of ZmPGP1

The *ZmPGP1* sequence alignment of 349 inbred lines, 68 landraces, and 32 teosintes spanned 9710 bp, which covered a 1762 bp upstream region, a 182 bp 5′UTR region, a 6821 bp coding region containing five exons and four introns, a 400 bp 3′UTR region, and a 545 bp downstream region (Table 1). Sequence polymorphisms, including SNPs and InDels, at *ZmPGP1* were identified, and 1070 variations were detected, including 878 SNPs and 192 InDels. On average, SNPs and InDels were found every 11.06 bp and 50.57 bp, respectively. The highest frequency of SNPs and InDels were found in the 3′UTR (5.86 bp) and 5′UTR (14 bp). The overall nucleotide diversity (π) of the *ZmPGP1* locus was 0.007. Among five regions of the *ZmPGP1*, and when the coding regions were less diverse than other regions (0.006), the downstream and 3′UTR showed high nucleotide diversity (0.016 and 0.015, respectively).

### 3.2. Nucleotide Diversity and Selection of ZmPGP1 in Inbred Lines, Landraces and Teosinte

**Figure 1:**
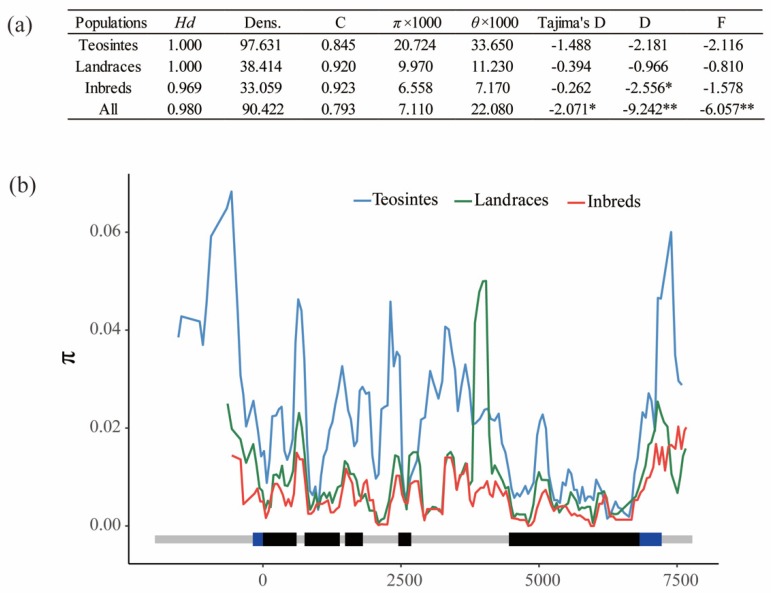
Nucleotide diversity in inbred lines, landraces, and teosinte. (**a**) Summary of nucleotide polymorphisms and neutrality test of ZmPGP1, Hd represents haplotype diversity, Dens denotes number of single nucleotide polymorphisms (SNP) per 1000 bp, C represents sequence conservation, and D* and F* represent Fu and Li’s D*and F*. (**b**) Nucleotide diversity (π) of inbred lines, landraces, and teosinte. π was calculated using the sliding windows method with a window size of 100 bp and a step length of 25 bp. * indicates a statistical significance at *p* < 0.05 level, ** indicates a statistical significance at *p* < 0.01 level.

To investigate the genetic diversity of *ZmPGP1* in inbred lines, landrace, and teosinte, the sequence conservation (C) and nucleotide diversity (π) were analyzed and compared. For all test lines, the values of C and π × 1000 were 0.793 and 7.110, respectively (Figure 1a). Compared with teosintes, landraces and inbred lines showed higher conservation (C_T_ = 0.845, C_L_ = 0.920 and C_I_ = 0.923) and lower diversity (π × 1000_T_ = 20.724, π × 1000_L_ = 9.970 and π × 1000_I_ = 6.558). The highest divergence between inbred lines and teosintes was observed in the upstream and downstream regions (Figure 1b). A divergence peak was found in the fourth intron by comparing landraces to inbred lines. To investigate the involvement in maize domestication and improvement of *ZmPGP1*, the entire sequence was tested by the neutral test, including Tajima’s D and the D* and F* of Fu and Li. The values for Tajima’s D and the D* and F* of Fu and Li of *ZmPGP1* were significantly less than 0, indicating this gene maybe selected in the domestication and improvement process (Figure 1a).

### 3.3. Association Analysis of Phenotypic Traits with ZmPGP1

**Figure 2:**
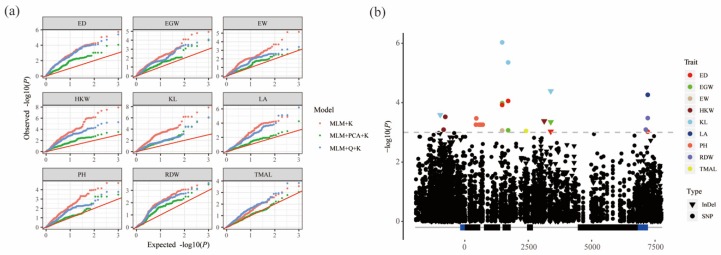
*ZmPGP1*-based association mapping. (**a**) QQ plot for the association analysis under three models, red, green and blue dots denote MLM + K, MLM + PCA + K, and MLM + Q + K, respectively. (**b**) Manhattan plot by using the MLM + PCA + K model. Triangles and dots represent InDels and SNPs, respectively. Abbreviations for traits are as follows: ED, ear diameter; EGW, ear grain weight; EW, ear weight; HKW, 100-kernel weight; KL, kernel length; LA, leaf angle; PH, plant height; RDW, root dry weight; TMAL, tassel main axis length.

To identify significant variants associated with phenotypic traits, association analysis was performed using 499 variants, including 269 SNPs and 230 InDels with minor allele frequency (MAF) ≥0.05 in 349 inbred lines. Three mixed linear models (MLM), MLM + K, MLM + Q + K, and MLM + PCA + K, were employed to perform marker-traits association analysis. Comparing the Quantile-Quantile (QQ) plots generated for these models, we selected MLM + PCA + K to minimize both false positives and false negatives (Figure 2a).

**Figure 3:**
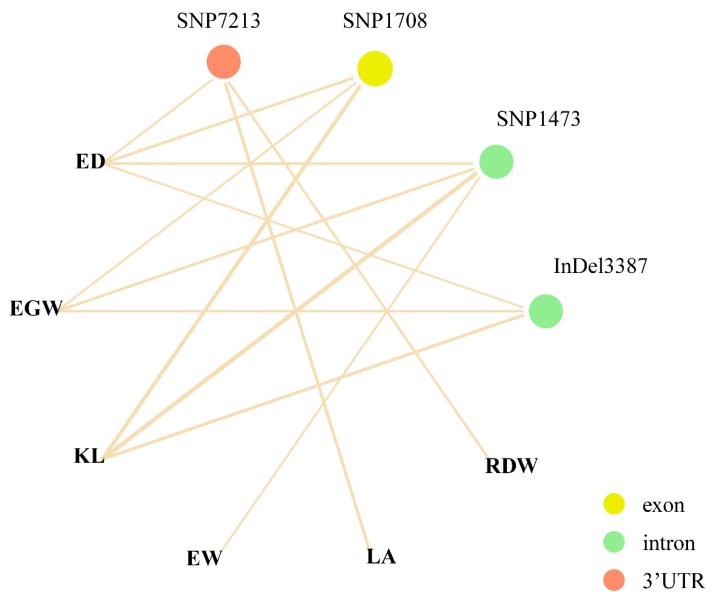
The network between pleiotropic site and associated traits. Yellow, green, and orange circle indicated the variations were in exon, intron, and 3′UTR, and the lines represent *p* value.

Using a Bonferroni correction based on 499 *ZmPGP1*-based markers, the *P*-value thresholds were set at 0.001 (0.5/499). A total of 24 significant marker–trait associations involved 15 variants (12 SNPs and 3 InDels) were identified for 9 traits using the MLM + PCA + K model (Table S1). Among these 24 associations, 9 and 15 sites were associated with 4 plant architecture (PH, LA, TMAL, and RDW [root dry weight]) and 5 ear traits (ED, EGW, EW, HKW and KL), respectively (Figure 2b; Table S2). A total of 3, 4, 6, and 2 variants were distributed in the upstream, exon, intron, and 3′UTR regions, respectively. The SNP at site 1708 in exon 3, which was associated with ED, EGW, and KL, caused synonymous changes. SNPs at sites 438, 453, and 555 in exon 1 caused non-synonymous changes in the amino acid sequence. Three high LD SNPs 438, 453, and 555 were associated with PH (Table S1). All significant variants could explain 2.98–6.91% of the phenotypic variation. Most of the associations were small effect variants and could explain less than 4% of the phenotypic variation. SNP1473, associated with KL (*p* = 9.34 × 10^−7^), explained the most phenotypic variation, up to 6.93%. In addition, 4 pleiotropic sites, including SNP1473, SNP1708, SNP7213, and InDel3387, were significantly associated with ED, EGW, KL, EGW, LA, and RDW (Figure 3). SNP1473 in intron 2 was associated with four ear traits (ED, EGW, KL, and EW), SNP1708 was associated with three ear traits (ED, EGW, and KL), SNP7213 in the 3′UTR was associated with ED, LA, and RDW, and the InDel at site 3387 in intron 4 was associated with ED, EGW, and KL (Table 2).

**Figure 4:**
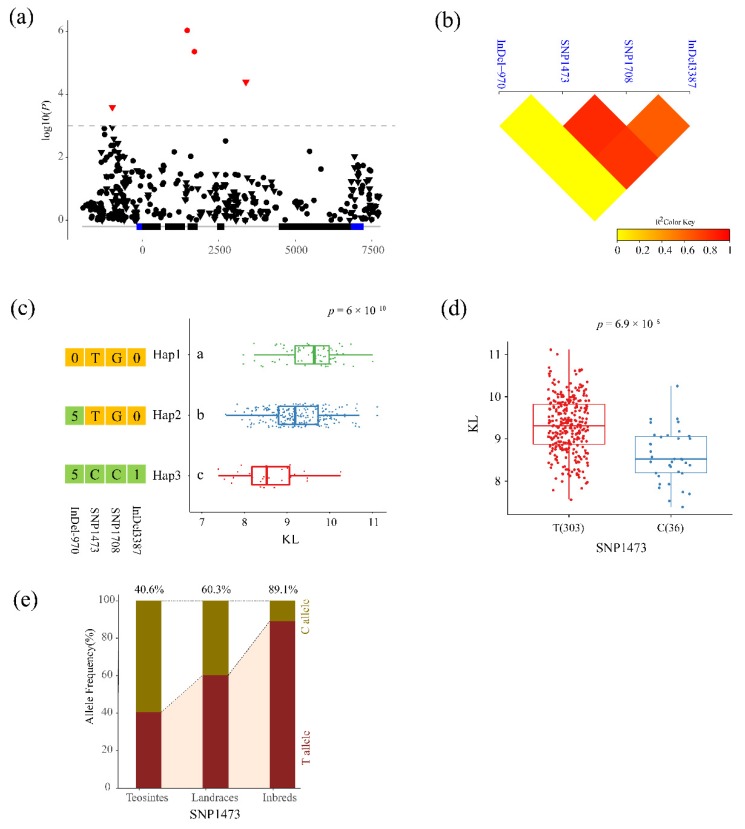
Natural variations in *ZmPGP1* were significantly associated with KL. (**a**) *ZmPGP1*-based association mapping for kernel length (KL). (**b**) Linkage disequilibrium (LD) heatmap for six significant variants associated with KL. (**c**) Haplotypes of *ZmPGP1* among natural variations in inbred lines. (**d**) Comparison of kernel length between different alleles of SNP1473. (**e**) The allele frequency of SNP1473 in teosinte, landraces, and inbred lines.

Linkage disequilibrium (LD) analysis found that SNP438, SNP453, SNP555, SNP628 and SNP706 showed strong LD (*r*^2^ > 0.95) with each other in inbred lines. After the clumping of variants, 11 significant sites were identified. Six major haplotypes which contained more than 10 lines emerged from the 11 significant sites across inbred lines, and a significant phenotypic difference was observed between haplotypes in 8 traits, except for TMAL (Table S2). Four significant variants were significantly associated with KL, including InDel-970, SNP1473, SNP1708, and InDel3387. Three major haplotypes, which contained more than 20 lines, emerged from the 4 significant sites across 349 inbred lines (Figure 4c). The phenotypic differences in KL between the three major haplotypes were compared, and a significant difference was detected by ANOVA (*p* = 6 × 10^−10^) between haplotypes. Hap1, carrying all increased alleles, had the longest kernel length, followed by Hap2, which included the majority of tested inbred lines. Hap3, carrying all decreased alleles, had the shortest kernel length. SNP1473 was the most significant sites, the allele T group had a significantly longer KL than the allele C group (*p* = 6.9 × 10^−8^, Figure 4d). Further, we analyzed the allele frequencies among the three populations. The results showed that the frequency of the SNP1473T in teosintes was 40.6%, and in landraces and inbred lines, the frequency increased to 60.3% and 89.1%, respectively (Figure 4e). These results suggested that SNP1473 might have been selected during domestication and improvement of maize. Three variants at sites 1473, 1708, and 3387 were significantly associated with EGW, which could divide the tested inbred lines into 2 major haplotypes (Figure S1). A significant difference between haplotypes was observed for EGW (*p* = 1.3 × 10^−4^). The SNP at site 1473 also had the most significant association with EGW. Three variants were identified for HKW that divided the inbred lines into four groups (Figure S2). The HKW of Hap1 was higher than the other three haplotypes (*p* = 8.3 × 10^−9^). The most significant site was SNP-769, and the frequency of the increased allele, SNP-769T, increased from 8.3% in teosintes to 33.3% in inbred lines. Five SNPs with high LD (*r*^2^ > 0.95) were significantly associated with PH. The plant height in the inbred lines carrying allele SNP453G was higher than those containing the C allele (Figure S3). The frequency of the G allele decreased from 50.0% in teosintes to 16.1% in inbred lines. Two SNPs significantly associated with RDW divided the tested inbred lines into 3 major haplotypes (Figure S4). The frequency of increased allele SNP7137C increased from 0 in teosintes to 74.1% in inbred lines.

## 4. Discussion

The process of maize domestication and improvement has been studied with population genetics–genomics [3], QTL mapping [38], and gene expression assays [39]. During domestication and improvement, the plant morphology and productivity of maize have changed dramatically. Maize plants typically have one or two short branches and only two ears, each with several hundred kernels [38]. These changes involved artificial selection of specific genes controlling key morphological and agronomic traits [40], resulting in reduced genetic diversity. Previous studies have identified several genes underlying maize evolution: 484 domestication and 695 improvement regions were identified from population genetics analyses [3]. It is estimated that approximately 2–4% of genes have been selected during maize domestication and improvement [40]. Here, we examined DNA sequence variation in *ZmPGP1*, which is involved in the polar movement of indole-3-acetic acid (IAA). Plant hormones, such as auxins, play a key role in plant growth, development, defenses, and stress tolerance [41]. A previous study reported that an auxin response factor might contribute to the morphological difference between maize and teosinte [40]. We found that the level of nucleotide diversity (π × 1000) in teosintes is 20.724, decreased to 9.970 in landraces and 6.558 in maize inbred lines (Figure 1a), suggesting that approximately half of the genetic diversity has been lost during domestication process. Similar results were observed in several plants, such as soybeans and cucumbers [42,43]. Many previous studies employed only a limited number of teosinte, landraces, and maize to identify the domestication signals. For example; a total of 14 inbred lines, 16 landraces, and 16 teosinte accessions were chosen to artificial selection of 1095 genes. 28 inbred lines, 16 landraces, and 16 teosinte accessions were used to investigate the involvement of 32 MADS-box genes during maize domestication and improvement [44,45]. In this study, a larger population including 349 inbred lines, 68 landraces and 32 wild relatives were used to re-sequence *ZmPGP1* with high sequencing depth (more than 100×), which could help us to identify the selection signals with larger effective and high accurate.

Plant architecture and kernel and ear traits, the key factors affecting grain yield, were the main traits targeted of maize breeding. The identification of the natural variations in these traits could help to improve the efficiency of breeding selection. Although hundreds of QTLs related to these traits have been identified [10,11], few genes have been cloned from the natural germplasm. *ZmPGP1* (ABCB1 or br2), involved in auxin polar transport, has been shown to be associated with plant height, stalk diameter, leaf length and leaf angle [28]. Three Mu insertions were detected in the exon and intron of *ZmPGP1* [16]. These mutations dramatically affected height reduction but were rare variations in natural accessions. Natural germplasm with a broad genetic base could be a potential resource for improving yield [46]. Natural variations of *ZmPGP1* have also been identified [16,27,28,29,30,31], and some alleles have great potential in maize improvement. One rare SNP variant in the exon could reduce plant height without affecting yield [47]. A new 241-bp deletion in the last exon of PGP1 also had no negative effect on yield, but significantly reduced plant height and ear height and increased stalk diameter and erected leaves. The deletion was a rare allele that could be detected in only one line of 311 diverse maize accessions [28]. The result revealed that *ZmPGP1* has good potential to reshape plant architecture without the loss of yield in maize breeding. Candidate gene association analysis can identify the elite variation and the best haplotype for target traits. The elite variations of more than 30 genes involved in flowering time, kernel composition, drought tolerance, and root development were detected by candidate gene association analysis [48]. For example, *crtRB1* was proved to be associated with β carotene concentration and conversion in maize kernels, and the most favorable alleles were developed to inexpensive markers to use for crop provitamin A biofortification [49]. In this study, to identify the natural variations and favorable haplotypes of *ZmPGP1*, 1070 variations were detected from 9710 bp re-sequenced genomic region of *ZmPGP1*. In total, 11 variants were identified for 5 yield-related traits and 4 plant architecture (Figure 2; Table 2). However, two previously rare variations [28,29,30,31,47] were not found in our study. SNP1473 was the most significant variant for KL and EGW. The frequency of the increased allele T was 40.6% in teosintes and was enriched to 60.3% and 89.1% during maize domestication and improvement (Figure 4; Figure S1). The selection patterns were similar with the 1.2-Kb presence-absence variant of *KRN4*, which is likely responsible for increased kernel row number in maize [15]. In conclusion, we re-sequenced the *ZmPGP1* gene in 349 inbred lines, 68 landraces, and 32 teosintes, sequence polymorphisms, nucleotide diversity and neutral tests revealed that *ZmPGP1* might be selected during domestication and improvement processes. A total of 11 variants significantly associated with 4 plant architecture and 5 ear traits were identified by marker–trait association analysis in inbred lines. The significant variants could be used to develop new markers to improve plant architecture and ear traits in maize.

## Figures and Tables

**Table 1:** Summary of parameters for the analysis of nucleotide polymorphisms of the maize genes *ZmPGP1.*

Parameters	Upstream	5′UTR	Coding Region	3′UTR	Downstream	Entire Region
Total length of amplicons (bp)	1762	182	6821	400	545	9710
Number of all of the sequence variants	69	32	779	86	104	1070
Frequency of all of the sequence variants	0.039	0.176	0.114	0.215	0.191	0.110
Number of nucleotide substitutions (bp)	43	19	663	67	86	878
Frequency of polymorphic sites per bp	0.024	0.104	0.097	0.168	0.158	0.090
Number of indels	26	13	116	19	18	192
Number of indels sites	34	42	445	36	73	640
Average indel length	1.308	3.231	3.836	1.895	4.056	3.333
Frequency of indels per bp	0.015	0.071	0.017	0.048	0.033	0.020
π × 1000	12.890	7.990	6.020	15.270	15.660	7.110
θ × 1000	40.870	22.720	19.110	36.120	53.510	22.080
Tajima’s D	−1.905 *	−1.620	−2.089 *	−1.659	−2.065 *	−2.071 *
Fu and Li’s D	−5.458 **	−2.149	−8.874 **	−8.232 **	−7.498 **	−9.242 **
Fu and Li’s F	−4.605 **	−2.342 *	−5.917 **	−6.037 **	−5.699 **	−6.057 **

* indicates a statistical significance at *p* < 0.05 level, ** indicates a statistical significance at *p* < 0.01 level. “UTR” indicated untranslated region.

**Table 2:** Significant markers associated with phenotypic traits.

Trait	Marker	Allele	*p* Value	−lg (*P*)	*R* ^2^	Region	Position ^a^
ED	SNP1473	**T**/C	1.20 × 10^−4^	3.92	4.74%	intron2	1473
ED	SNP1708	**G**/C	8.75 × 10^−5^	4.06	4.93%	exon3	1708
ED	InDel3387	**-**/G	9.34 × 10^−4^	3.03	3.49%	intron4	3387
ED	SNP7213	**T**/A	9.52 × 10^−4^	3.02	3.47%	3′UTR	7213
EGW	SNP1473	**T**/C	1.04 × 10^−4^	3.98	4.06%	intron2	1473
EGW	SNP1708	**G**/C	8.53 × 10^−4^	3.07	2.98%	exon3	1708
EGW	InDel3387	**-**/G	4.47 × 10^−4^	3.35	3.31%	intron4	3387
EW	SNP1473	**T**/C	8.64 × 10^−4^	3.06	3.28%	intron2	1473
HKW	SNP-769	C/**T**	3.01 × 10^−4^	3.52	4.12%	upstream	−769
HKW	SNP-836	**C**/A	8.11 × 10^−4^	3.09	3.19%	upstream	−836
HKW	InDel3129	T/**-**	4.17 × 10^−4^	3.38	3.42%	intron4	3129
KL	InDel-970	GACAG/**-----**	2.58 × 10^−4^	3.59	3.78%	upstream	−970
KL	SNP1473	**T**/C	9.34 × 10^−7^	6.03	6.91%	intron2	1473
KL	SNP1708	**G**/C	4.42 × 10^−6^	5.36	6.03%	exon3	1708
KL	InDel3387	**-**/G	4.06 × 10^−5^	4.39	4.79%	intron4	3387
LA	SNP7213	T/**A**	5.44 × 10^−5^	4.26	3.94%	3′UTR	7213
PH	SNP453	C/**G**	3.38 × 10^−4^	3.47	3.21%	exon1	453
RDW	SNP7137	**C**/G	8.07 × 10^−4^	3.09	4.21%	3′UTR	7137
RDW	SNP7213	**T**/A	3.30 × 10^−4^	3.48	4.85%	3′UTR	7213
TMAL	SNP2414	**G**/A	8.94 × 10^−4^	3.05	4.36%	intron3	2414

^a^ The position of the start codon (ATG) is labelled as “0”.

## Supplementary Materials

The following are available online at https://www.mdpi.com/2073-4425/10/9/664/s1, Table S1: All significant markers associated with phenotypic traits. Table S2: Phenotypic differences among different haplotypes. Figure S1: Natural variations in *ZmPGP1* were significantly associated with EGW. Figure S2: Natural variations in *ZmPGP1* were significantly associated with HKW. Figure S3: Natural variations in *ZmPGP1* were significantly associated with PH. Figure S4: Natural variations in *ZmPGP1* were significantly associated with RDW. Dataset 1: The sequence of ZmHKT1.fa. Dataset 2: The variaton of ZmPGP1.fa.
