# Supplementary material for: Natural Variation and Domestication Selection of ZmPGP1 Affects Plant Architecture and Yield-Related Traits in Maize
Source: Genes (Basel). 2019 Aug 30;10(9):664. doi: 10.3390/genes10090664 (PMC6770335; doi:10.3390/genes10090664)
Supplement: Supplementary file 1 [file genes-10-00664-s001.zip › Supplementary Materials/Supplementary Table and Figure .docx]

**Supplementary Table**

**Table S1** All significant markers associated with phenotypic traits

**Table S2** Phenotypic differences among different haplotypes.

**Supplementary Figure**

**Figure S1** Natural variations in *ZmPGP1* were significantly associated with EGW

**Figure S2** Natural variations in *ZmPGP1* were significantly associated with HKW

**Figure S3** Natural variations in *ZmPGP1* were significantly associated with PH

**Figure S4** Natural variations in *ZmPGP1* were significantly associated with RDW

**Table S1.** All significant markers associated with phenotypic traits.

| Trait | Marker | Allele | *P* value | -lg(*P*) | R^2^ | Region | Position |
| --- | --- | --- | --- | --- | --- | --- | --- |
| ED | SNP1473 | **T**/C | 1.20E-04 | 3.92 | 4.74% | intron2 | 1473 |
| ED | SNP1708 | **G**/C | 8.75E-05 | 4.06 | 4.93% | exon3 | 1708 |
| ED | InDel3387 | **-**/G | 9.34E-04 | 3.03 | 3.49% | intron4 | 3387 |
| ED | SNP7213 | **T**/A | 9.52E-04 | 3.02 | 3.47% | 3’UTR | 7213 |
| EGW | SNP1473 | **T**/C | 1.04E-04 | 3.98 | 4.06% | intron2 | 1473 |
| EGW | SNP1708 | **G**/C | 8.53E-04 | 3.07 | 2.98% | exon3 | 1708 |
| EGW | InDel3387 | **-**/G | 4.47E-04 | 3.35 | 3.31% | intron4 | 3387 |
| EW | SNP1473 | **T**/C | 8.64E-04 | 3.06 | 3.28% | intron2 | 1473 |
| HKW | SNP-769 | C/**T** | 3.01E-04 | 3.52 | 4.12% | upstream | -769 |
| HKW | SNP-836 | **C**/A | 8.11E-04 | 3.09 | 3.19% | upstream | -836 |
| HKW | InDel3129 | T/**-** | 4.17E-04 | 3.38 | 3.42% | intron4 | 3129 |
| KL | InDel-970 | GACAG/**-----** | 2.58E-04 | 3.59 | 3.78% | upstream | -970 |
| KL | SNP1473 | **T**/C | 9.34E-07 | 6.03 | 6.91% | intron2 | 1473 |
| KL | SNP1708 | **G**/C | 4.42E-06 | 5.36 | 6.03% | exon3 | 1708 |
| KL | InDel3387 | **-**/G | 4.06E-05 | 4.39 | 4.79% | intron4 | 3387 |
| LA | SNP7213 | T/**A** | 5.44E-05 | 4.26 | 3.94% | 3’UTR | 7213 |
| PH | SNP438 | C/**T** | 5.51E-04 | 3.26 | 2.98% | exon1 | 438 |
| PH | SNP453 | C/**G** | 3.38E-04 | 3.47 | 3.21% | exon1 | 453 |
| PH | SNP555 | C/**T** | 5.51E-04 | 3.26 | 2.98% | exon1 | 555 |
| PH | SNP628 | C/**T** | 5.51E-04 | 3.26 | 2.98% | intron1 | 628 |
| PH | SNP706 | C/**A** | 5.51E-04 | 3.26 | 2.98% | intron1 | 706 |
| RDW | SNP7137 | **C**/G | 8.07E-04 | 3.09 | 4.21% | 3’UTR | 7137 |
| RDW | SNP7213 | **T**/A | 3.30E-04 | 3.48 | 4.85% | 3’UTR | 7213 |
| TMAL | SNP2414 | **G**/A | 8.94E-04 | 3.05 | 4.36% | intron3 | 2414 |

**Table S2** Phenotypic differences among different haplotypes.

| Haplotype | ED | EGW | EW | HKW | KL | LA | PH | RDW | TMAL | Counts |
| --- | --- | --- | --- | --- | --- | --- | --- | --- | --- | --- |
| Hap1 | 3.96a | 61.26a | 73.35ab | 24.70b | 9.69a | 30.06b | 176.66a | 90.43ab | 26.33a | 30 |
| Hap2 | 3.78b | 57.66a | 70.29ab | 24.25bc | 9.31b | 29.24b | 168.31b | 86.45abc | 26.84a | 62 |
| Hap3 | 3.80ab | 54.47a | 65.73b | 22.46c | 8.79c | 29.20bc | 168.53ab | 70.23c | 25.89a | 11 |
| Hap4 | 3.78ab | 56.05a | 68.90ab | 23.64bc | 9.37ab | 33.76a | 179.26a | 76bc | 27.58a | 15 |
| Hap5 | 3.92a | 61.11a | 74.33a | 26.20a | 9.44ab | 28.71bc | 177.10a | 94.62a | 26.10a | 51 |
| Hap6 | 3.95a | 61.55a | 73.25ab | 27.41a | 9.45ab | 26.18c | 159.41b | 92.76ab | 26.71a | 26 |

Different letters within each column indicate significant difference (P<0.05).

**
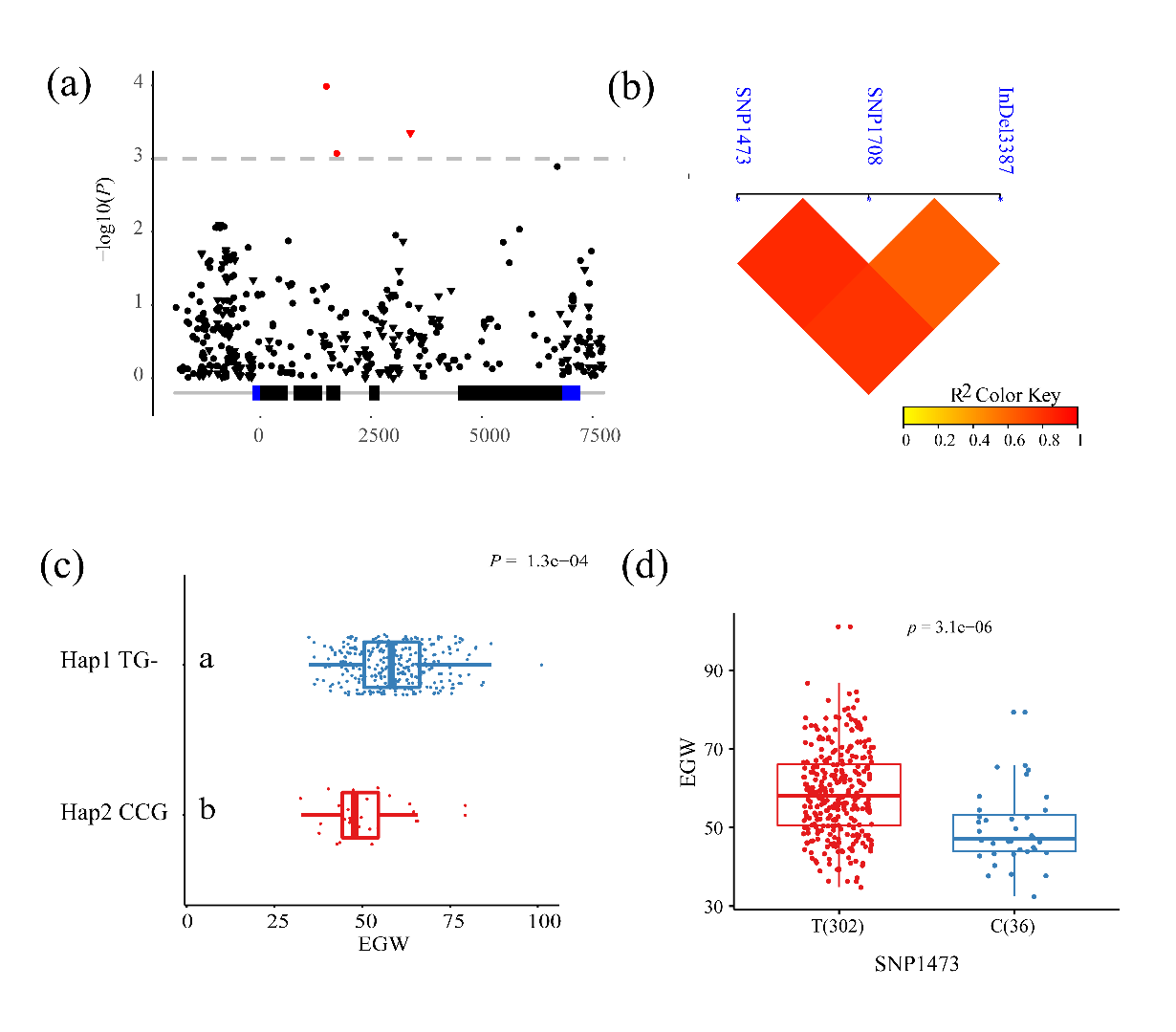
**

**Figure S1.** Natural variations in ZmPGP1 were significantly associated with EGW.

(**a**) ZmPGP1-based association mapping for EGW. (**b**) Linkage disequilibrium (LD) heatmap for three significant variants associated with EGW. (**c**) Haplotypes of *ZmPGP1* among natural variations in inbred lines. (**d**) Comparison of EGW between different alleles of SNP1473.


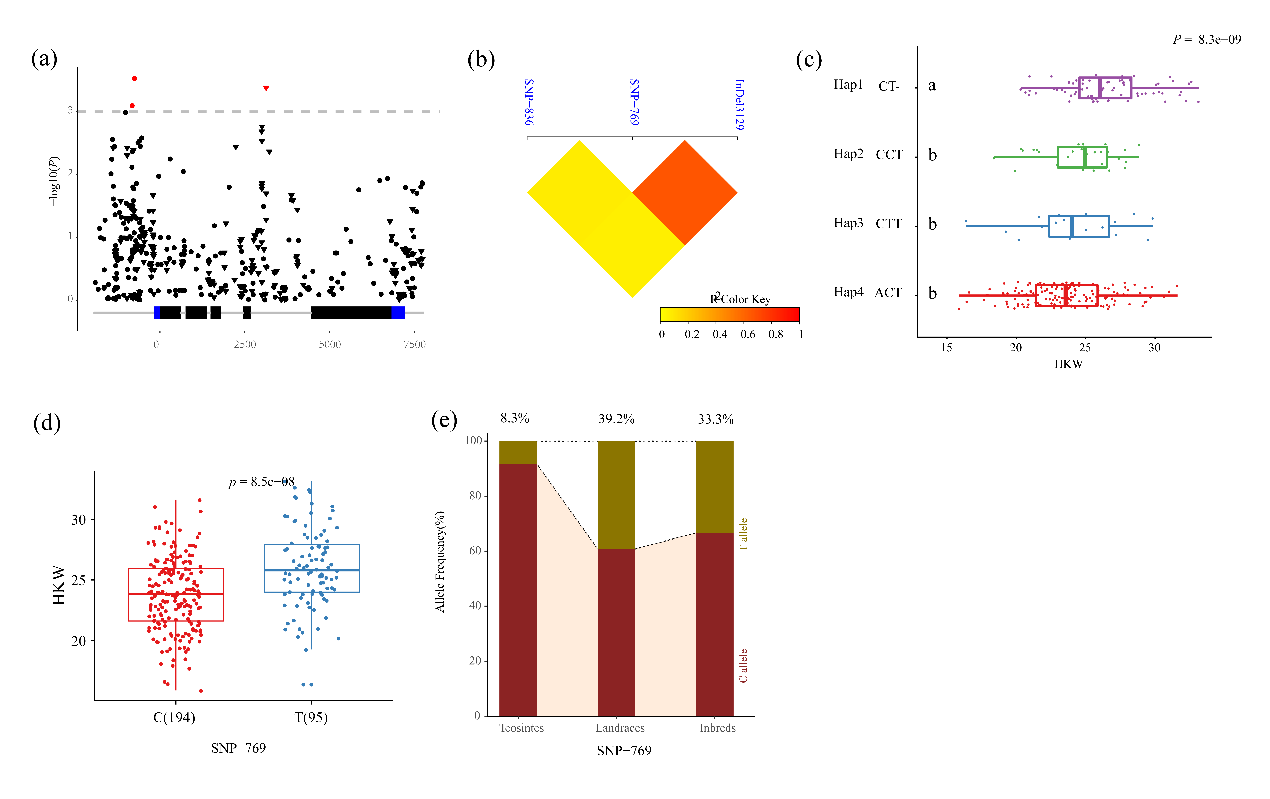


**Figure S2.** Natural variations in ZmPGP1 were significantly associated with HKW.

(**a**) ZmPGP1-based association mapping for HKW. (**b**) Linkage disequilibrium (LD) heatmap for three significant variants associated with HKW. (**c**) Haplotypes of *ZmPGP1* among natural variations in inbred lines. (**d**) Comparison of HKW between different alleles of SNP-769. (**e**) The allele frequency of SNP-769 in teosinte, landraces and inbred lines.


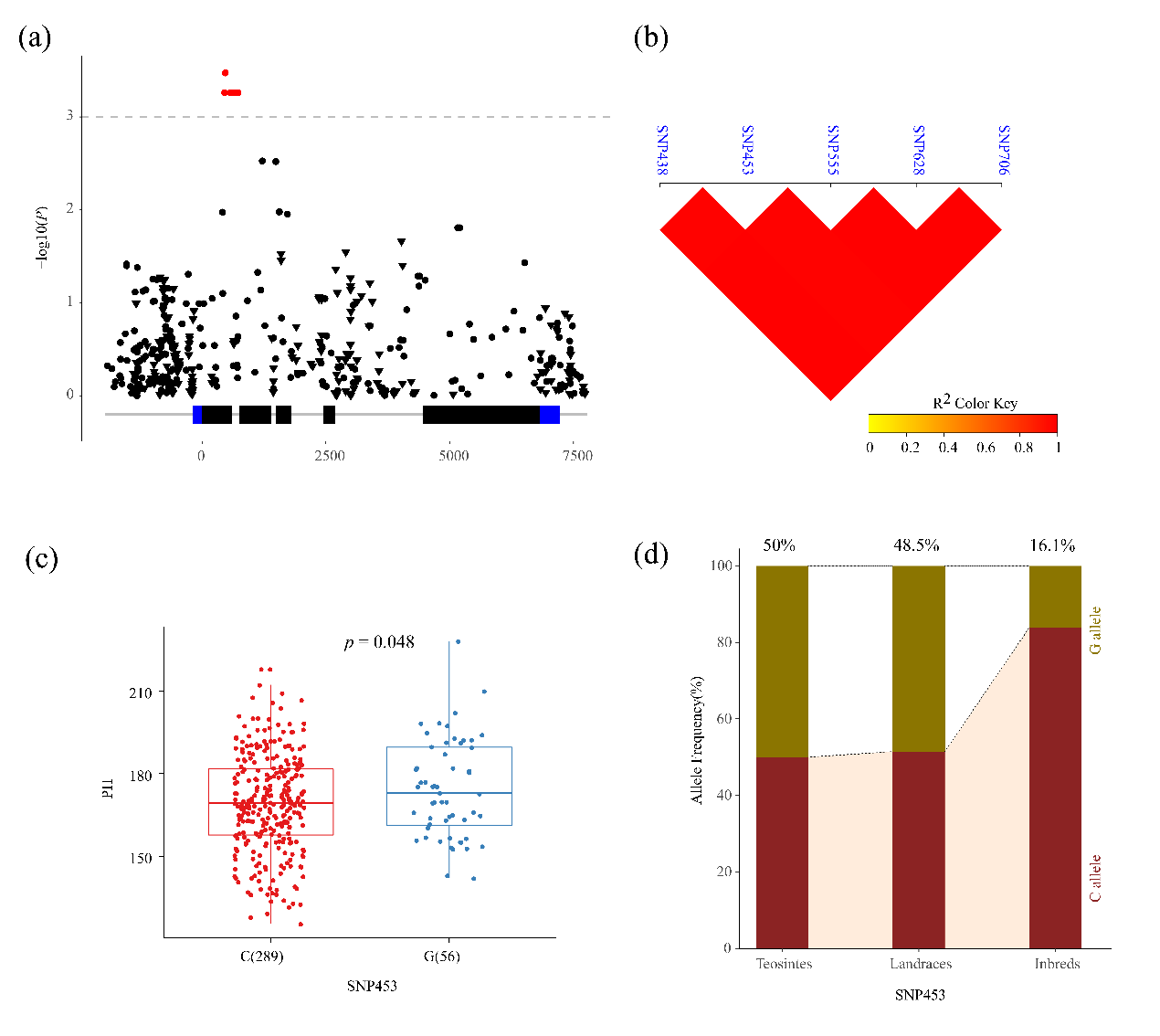


**Figure S3.** Natural variations in ZmPGP1 were significantly associated with PH.

(**a**) ZmPGP1-based association mapping for PH. (**b**) Linkage disequilibrium (LD) heatmap for five significant variants associated with PH. (**c**) Comparison of PH between different alleles of SNP453. (**d**) The allele frequency of SNP453 in teosinte, landraces and inbred lines.


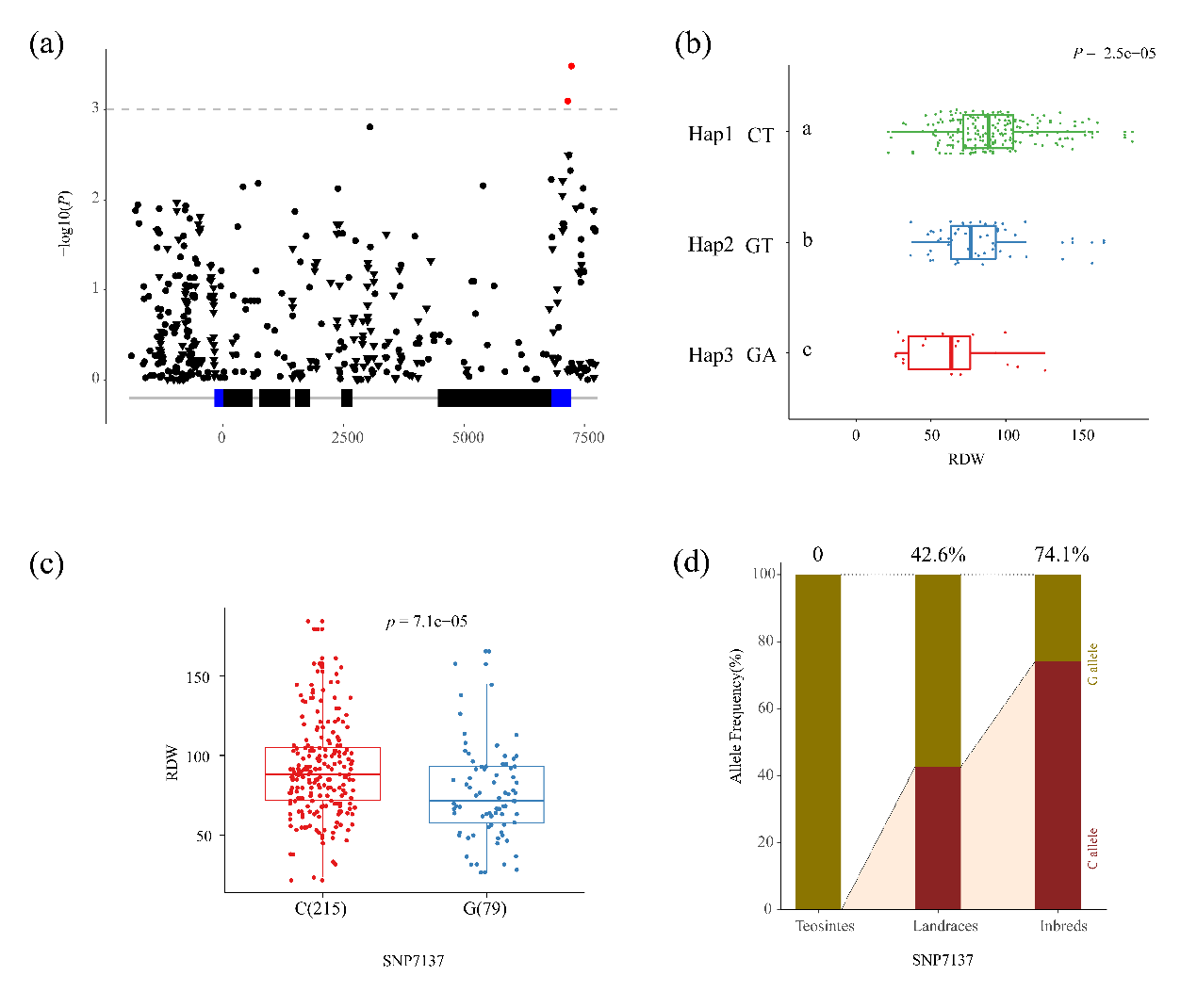


**Figure S4.** Natural variations in ZmPGP1 were significantly associated with RDW.

(**a**) ZmPGP1-based association mapping for RDW. (**b**) Haplotypes of *ZmPGP1* among natural variations in inbred lines. (**d**) Comparison of RDW between different alleles of SNP7137. (**e**) The allele frequency of SNP7137 in teosinte, landraces and inbred lines.
